# Diseases Admitted under the Care of the Physicians of the Westminster Hospital, from the 18th of Feb. to the 20th of March, 1800

**Published:** 1800-04

**Authors:** 


					State of Difeafes in London. 303
Difeafes admitted under the Care of the Phyjtciam ? of the Wejimitffter
Jfojpital, from the iSth of Feb: to the zoth of March, 18oo.
Continued Fever - 10
Quotidian - - - i
Fleurify ? - - 3
Amenorrhcea - - $
Anafarca - 5
Afcites - - - 3
Afthenia
Afthenia - - - '4
Afthma - - 5
Catarrh - - , 2
Cholera' 1
Chorea ? - - 1
Cough * ^ - - 16
Diarrhoea'x - - 5
Dyfentery 1
Dyfpricea" - 7
Dyfuria - - - 1
v Enterodynia ' - - 6
Epilepfy ? 2
* Haemoptyfis - 2
Hapmorrhois ' - I
Hypochondriacs - i
Impetigo - - 2
Leucorrhaea - j- - I
Menorrhagia - - i
Obftipatio i
Paralyfis - - I
Phthifis - z
Pleurodynia ' - - i
Prurigo - - - - i
Rheumatifms - - it
Struma - 4
Syphilis - - - * I
Scirrhus - - I
Worms - * 2

				

